# Dietary exposure to *N*-nitrosamines and their precursors: an age-stratified assessment

**DOI:** 10.1038/s41598-025-34144-7

**Published:** 2026-01-19

**Authors:** Aida Zapico, David Herrero-Morin, Silvia Arboleya, Clara G. de los Reyes-Gavilán, Miguel Gueimonde, Sonia González

**Affiliations:** 1 Department of Functional Biology, University of Oviedo, Oviedo, 33006 Spain; 2 Diet, Microbiota and Health Group, Instituto de Investigación Sanitaria del Principado de Asturias (ISPA), Oviedo, 33011 Spain; 3 Pediatrics Service, Centro Atención Primaria Infiesto, SESPA, Piloña, 33530 Spain; 4 Department of Microbiology and Biochemistry of Dairy Products, Instituto de Productos Lácteos de Asturias (IPLA-CSIC), Oviedo, 33011 Spain

**Keywords:** *N*-nitrosamines (*N*-NAs), Nitrate, Nitrite, Endogenous nitrosation, Risk assessment, Vulnerable populations, Cancer, Diseases, Environmental sciences, Health care, Medical research, Oncology, Risk factors

## Abstract

Dietary exposure to *N*-nitrosamines (*N*-NAs) may pose health concerns across all age groups, particularly in children and the elderly, due to their association with increased cancer risk. This study risk-assessed age-specific dietary intake of *N*-NAs and main dietary sources. *N*-NAs consumption was estimated from 671 questionnaires (validated Food Frequency Questionnaires) obtained from 116 children (followed-up from 6 months to 4 years) and 182 adults (aged 19 to 95 years) from northern Spain (Asturias). The content of nitrates, nitrites and *N*-NAs was calculated per 100 g of food for 348 items primarily using EFSA and EPIC data. Intakes were standardized by body weight and dietary sources were analysed using the FoodSources Shiny-app. The highest mean *N*-NAs intake, mainly of *N*-Nitrosodimethylamine (NDMA), was observed at 48 months and was derived from processed meats. Among adults, nitrite intake was primarily attributable to animal sources and 12 to 19% of individuals exceeded the acceptable daily intake (ADI). In children, the lowest margin of exposure (MOE) for *N*-NAs was found at 48 months, and MOEs below 10,000 were observed from 12 months to 95 years. Early childhood appears to be a critical window for reducing exposure to carcinogens, supporting the development of targeted public-health policies.

**Supplementary Information:**

The online version contains supplementary material available at 10.1038/s41598-025-34144-7.

## Introduction

In January 2023, the European Food Safety Authority (EFSA) published the findings on public health implications associated with the presence of *N*-nitrosamines (*N*-NAs) in the food supply^1^. The results demonstrated that current dietary exposure levels may present health concerns across all age groups within the European Union population^1^.

*N*-NAs, classified as probable human carcinogens by the International Agency for Research on Cancer (IARC)^2^, can be ingested through various dietary sources, particularly processed meats, cured products, and certain beverages^3–5^. Within food products, *N*-NAs are formed through nitrosation reactions which typically occurs during high-temperature cooking, curing, smoking, or fermentation of meats^6^. To date, approximately 15 different *N*-NAs have been identified in meat derivatives such as sausages, salami, and bacon^7^. The specific types and concentrations of *N*-NAs in food are influenced by multiple factors, including meat type, the presence of amino and carbonyl precursors, storage duration, and processing conditions such as temperature, pH, smoking intensity, and fermentation processes^7,8^.

However, it is estimated that up to 97% of total human exposure to *N*-NAs results from endogenous nitrosation^9,10^, a process in which dietary nitrate is reduced to nitrite—primarily by oral and gut microbiota—which subsequently reacts with amines to form *N*-NAs^11^. Nitrates are predominantly ingested via vegetables, especially leafy greens, with their concentrations heavily influenced by nitrate levels in irrigation water and the use of nitrogen-based fertilizers^12^. In contrast, dietary nitrite is largely derived from processed meats, where it serves as a preservative and colour fixative, although small amounts also occur naturally in vegetables^13,14^. Although both nitrates and nitrites are recognized precursors of endogenous nitrosation, this process can be either inhibited or enhanced depending on co-occurring dietary factors such as antioxidants as vitamin C or haem iron^15^. Accordingly, a higher risk of colorectal cancer has been reported when higher intakes of nitrates were consumed with lower vitamin C (83.9 mg/d) or higher red meats (≥ 43.5 g/d), whereas no association was observed for overall nitrate consumption^16^. These findings support the source-specific effect of nitrates and nitrites on health depicted by previous authors^17^, which may be potentially explained by *N*-NAs formation.

Long-term dietary intake of *N*-NAs has been positively associated with increased risk of several diseases. For example, the intake of *N*-Nitrosodimethylamine (NDMA), one of the most abundant *N*-NAs in foods^3^, has been associated with an increased risk of gastric or oesophageal cancers (for each 0.05–0.1 µg/d), as well as colorectal (2.29 vs. 0.03 µg/d) and pancreatic (0.12 vs. 0.04 µg/d) tumours^18–21^. These *N*-Nitroso compounds (NOCs) exhibit organotropism and induce DNA damage through alkylation mechanisms^22,23^, reinforcing the importance of minimizing dietary exposure across all age groups. This is particularly relevant for vulnerable groups such as children, who present a significantly higher exposure per kilogram of body weight (kg bw)^24^ and where long-term cumulative effects are a risk for future health. Also, although to lower extend, older adults constitute another vulnerable group since increasing the lifetime cumulative dose of *N*-NAs may increase carcinogenic potency^25^, suggesting potential age-related vulnerabilities. For NDMA, the estimated dietary intake ranges from 0.02 to 0.08 µg/d for Finnish children and adults, respectively (Penttilä et al., 1990), while Dutch exposure assessments show long-term 95th percentile (P95) dietary exposures of approximately 4 and 0.4 ng/kg bw/d for children and adults through vegetables and fish^26^.

Based on the potential risk derived from their intake, EFSA has established acceptable daily intake (ADI) values of 3.7 mg/kg bw/d for nitrate and 0.06 mg/kg bw/d for nitrite, while the U.S. EPA’s oral reference doses (RfD) are slightly more conservative (1.6 mg/kg bw/d for nitrate and 0.1 mg/kg bw/d for nitrite)^27–29^. For *N*-NAs, the margin of exposure (MOE) approach is commonly used to assess the risk derived from their intake^30^. This method compares the predicted dietary exposure to genotoxic compounds with the dose observed in experimental animals that results in a defined incidence of tumour formation. For this comparison, the MOE can be calculated based on the Benchmark Dose Lower Confidence Limit at 10% response (BMDL10), which represents the lowest dose associated with a 10% increase in tumour incidence, typically with a 95% confidence level. For genotoxic and carcinogenic substances, MOE values of 10,000 or higher based on the BMDL10, are considered of low concern from a public health point of view^31^. For NDMA, the BMDL10 derived from rat liver carcinogenicity studies has been reported as 35 µg/kg bw/d^1^ and MOE values lower than 10,000 for this *N*-NA and others were noted across all age groups^1^, raising potential concerns for public health.

The risk assessment of *N*-NA dietary exposure in the Spanish population is limited, particularly in vulnerable groups such as children and the elderly. Accordingly, this study aims to assess the intake of these compounds throughout life and to identify their main dietary sources in a sample population from Asturias, northern Spain. The results obtained would be essential for guiding the implementation of evidence-based nutritional interventions.

**Table 1:** General characteristics of the sample of study across age groups.

	6m *N* = 112	12m *N* = 109	24m *N* = 93	36m *N* = 84	48m *N* = 42	18-50y *N* = 21	51-65y *N* = 67	66-95y *N* = 94
Age (y)	0.50	1.00	2.00	3.00	4.00	42.86 ± 10.45	59.42 ± 4.16	74.83 ± 7.86
Gender								
Male	60 (54)	60 (55)	52 (56)	46 (55)	27 (64)	7 (33)	23 (34)	34 (36)
Weight (kg)	7.45 ± 1.06	9.72 ± 1.53	12.37 ± 1.77	14.86 ± 2.57	18.02 ± 3.48	71.45 ± 17.02	74.61 ± 13.98	71.74 ± 13.56
BMI								
(Z-score)	-0.42 ± 1.09	0.02 ± 1.35	0.05 ± 1.16	0.43 ± 1.29	0.00 ± 1.74	-	-	-
(kg/m^2^)	-	-	-	-	-	25.18 ± 4.43	26.87 ± 4.05	27.47 ± 4.34
Underweight	34 (32)	34 (33)	31 (36)	29 (36)	9 (22)	1 (5)	0 (0)	0 (0)
Normalweight	62 (59)	59 (58)	48 (55)	45 (56)	27 (66)	12 (57)	22 (33)	25 (27)
Overweight	8 (8)	8 (8)	8 (9)	6 (8)	5 (12)	4 (19)	33 (49)	46 (49)
Obese	1 (1)	1 (1)	0 (0)	0 (0)	0 (0)	4 (19)	12 (18)	23 (24)
Lifestyle								
Alcohol consumption	-	-	-	-	-	19 (90)	62 (93)	81 (86)
Current smoker	-	-	-	-	-	3 (14)	7 (13)	5 (14)
Practice of sport	-	-	-	-	-	10 (48)	14 (25)	11 (30)
Clinical history	-	-	-	-	-			
Hypertension	-	-	-	-	-	1 (7)	3 (9)	7 (30)
Diabetes	-	-	-	-	-	0 (0)	3 (9)	3 (13)
Asthma or allergies	-	-	-	-	-	2 (14)	8 (25)	2 (9)
Blood parameters (mg/dL)								
Glucose	-	-	-	-	-	-	94.6 ± 22.1	106.2 ± 29.7
Cholesterol	-	-	-	-	-	-	241.4 ± 41.1	209.0 ± 44.8
Triglycerides	-	-	-	-	-	-	95.8 ± 42.0	112.9 ± 47.4

Data is expressed as mean ± SD and N (%). Only respondents with available data were included in percentage calculations. (-) not applicable. BMI, body mass index

## Results

### Description of the sample

The general characteristics of the sample of study are presented in Table 1. Overweight or obesity was registered in a 67 and 73% of participants in the 51–65 and 66–95-age groups, respectively. In these age groups, 13–14% were current smokers and up to 93% reported alcohol consumption. The prevalence of hypertension, diabetes, and asthma or allergies in all adult groups was up to 30, 13 and 25%, respectively.

### Dietary intake across age groups

Energy and food intake across age groups is shown in Table 2. Human breast milk and infant products were the major contributors to total food intake at 6 months of age. Among children, those aged 48 months, exhibited the highest consumption of meat products (80 g/d), primarily processed meats (34 g/d). The highest intake of red meat was observed in adults aged 18–50 and 66–95 years (50 g/d). Additionally, the 18–50 age group showed the highest consumption of cereals and derivatives (222 g/d), legumes (65 g/d) and sauces and condiments (12 g/d), whereas among adults, the 66–95 age group presented the greatest intake of fish (70 g/d) and fruits (230 g/d).

**Table 2:** Energy and food group intake in the sample of study across age groups.

(g/d)	6m *N* = 112		12m *N* = 109	24m *N* = 93	36m *N* = 84	48m *N* = 42	18-50y *N* = 21	51-65y *N* = 67	66-95y *N* = 94
Energy (Kcal/d)	742.87 ± 340.33		1063.82 ± 218.67	1107.96 ± 271.97	1193.80 ± 301.00	1448.48 ± 360.66	2044.60 ± 729.48	1925.38 ± 664.96	2011.69 ± 614.03
Human breast milk (mL/d)	245.76 ± 338.75		111.38 ± 223.96	0 ± 0	0 ± 0	0 ± 0	-	-	-
Infant products	551.21 ± 367.65		340.16 ± 251.08	66.69 ± 146.16	39.14 ± 119.57	1.64 ± 6.49	-	-	-
Cereals and cereal products	4.05 ± 8.78		50.73 ± 32.49	74.52 ± 40.89	90.50 ± 48.64	106.10 ± 47.50	221.64 ± 196.00	178.92 ± 113.18	153.76 ± 81.38
Milk and dairy products	6.22 ± 27.52		164.89 ± 167.77	421.82 ± 206.24	384.95 ± 214.74	370.45 ± 208.24	302.54 ± 201.98	386.15 ± 243.14	457.45 ± 269.71
Meat and meat products	5.80 ± 15.03		38.22 ± 30.94	45.42 ± 30.77	53.27 ± 33.83	80.39 ± 42.45	172.18 ± 113.94	151.44 ± 125.00	137.50 ± 92.67
White meat	3.45 ± 8.49		18.80 ± 20.29	15.33 ± 17.09	16.77 ± 14.98	18.02 ± 19.14	62.50 ± 52.38	42.57 ± 33.73	35.56 ± 27.00
Red meat	2.33 ± 7.89		13.79 ± 14.34	15.93 ± 13.40	19.54 ± 19.95	18.92 ± 16.88	50.10 ± 39.02	46.42 ± 47.27	49.84 ± 53.07
Processed meat	0.03 ± 0.27		5.22 ± 6.46	14.15 ± 11.12	16.55 ± 11.47	33.80 ± 17.17	59.56 ± 53.78	61.38 ± 72.06	41.08 ± 29.55
Eggs	0.16 ± 1.73		13.34 ± 9.92	18.76 ± 10.55	21.12 ± 10.51	23.02 ± 10.66	42.44 ± 33.69	41.84 ± 25.10	30.77 ± 21.83
Fish	0.37 ± 1.75		33.66 ± 33.33	40.51 ± 28.40	42.88 ± 26.54	41.10 ± 31.70	39.80 ± 32.36	59.51 ± 32.50	69.71 ± 43.39
Seafood	0 ± 0		0 ± 0	1.54 ± 7.35	0.48 ± 2.53	2.44 ± 6.52	18.41 ± 16.68	19.42 ± 18.41	11.89 ± 18.53
Oils and fats	3.30 ± 4.91		10.50 ± 3.04	12.69 ± 5.34	17.44 ± 10.16	30.00 ± 17.53	17.82 ± 10.15	20.47 ± 17.26	27.91 ± 15.6
Vegetables	32.59 ± 47.32		160.80 ± 103.65	103.02 ± 86.99	106.11 ± 108.87	91.6 ± 104.69	278.31 ± 169.32	241.30 ± 152.51	289.68 ± 171.85
Legumes	0.81 ± 4.96		19.74 ± 20.48	34.43 ± 27.99	41.24 ± 37.34	36.31 ± 38.37	65.12 ± 108.44	37.47 ± 53.65	45.34 ± 56.24
Potatoes and tubers	60.94 ± 390.33		69.57 ± 72.68	44.57 ± 32.28	51.18 ± 48.58	67.48 ± 53.00	59.13 ± 31.16	48.30 ± 43.29	86.25 ± 70.14
Fruits	122.37 ± 139.30		235.61 ± 143.86	227.90 ± 165.98	220.84 ± 111.62	197.20 ± 116.69	120.54 ± 75.06	142.45 ± 85.28	229.81 ± 201.26
Nuts and seeds	0 ± 0		0 ± 0	0 ± 0	0 ± 0	0.32 ± 2.08	11.95 ± 14.05	14.73 ± 19.13	6.60 ± 14.83
Sugar and sweets	0.04 ± 0.38		0.07 ± 0.54	0.65 ± 1.59	1.11 ± 2.35	3.88 ± 4.10	13.03 ± 17.30	13.00 ± 16.53	9.62 ± 14.22
Snacks	0 ± 0		0 ± 0	0 ± 0	0 ± 0	0.52 ± 1.86	1.57 ± 2.87	1.70 ± 4.40	1.27 ± 4.24
Sauces and condiments	0 ± 0		0 ± 0	0.06 ± 0.36	0.05 ± 0.35	6.07 ± 5.68	11.73 ± 10.66	8.07 ± 6.18	4.86 ± 4.86
Non-alcoholic b. (mL/d)	0 ± 0		0 ± 0	5.73 ± 18.11	15.23 ± 40.12	42.48 ± 47.97	277.17 ± 217.90	234.82 ± 224.45	162.72 ± 155.98
Alcoholic b. (mL/d)	0 ± 0		0 ± 0	0 ± 0	0 ± 0	0.31 ± 1.26	128.40 ± 164.61	135.18 ± 186.22	99.28 ± 187.75

Data is expressed as mean ± SD. (-) not applicable. b. beverages

### Evolution of the intake of *N*-NAs across age groups

**Fig. 1:**
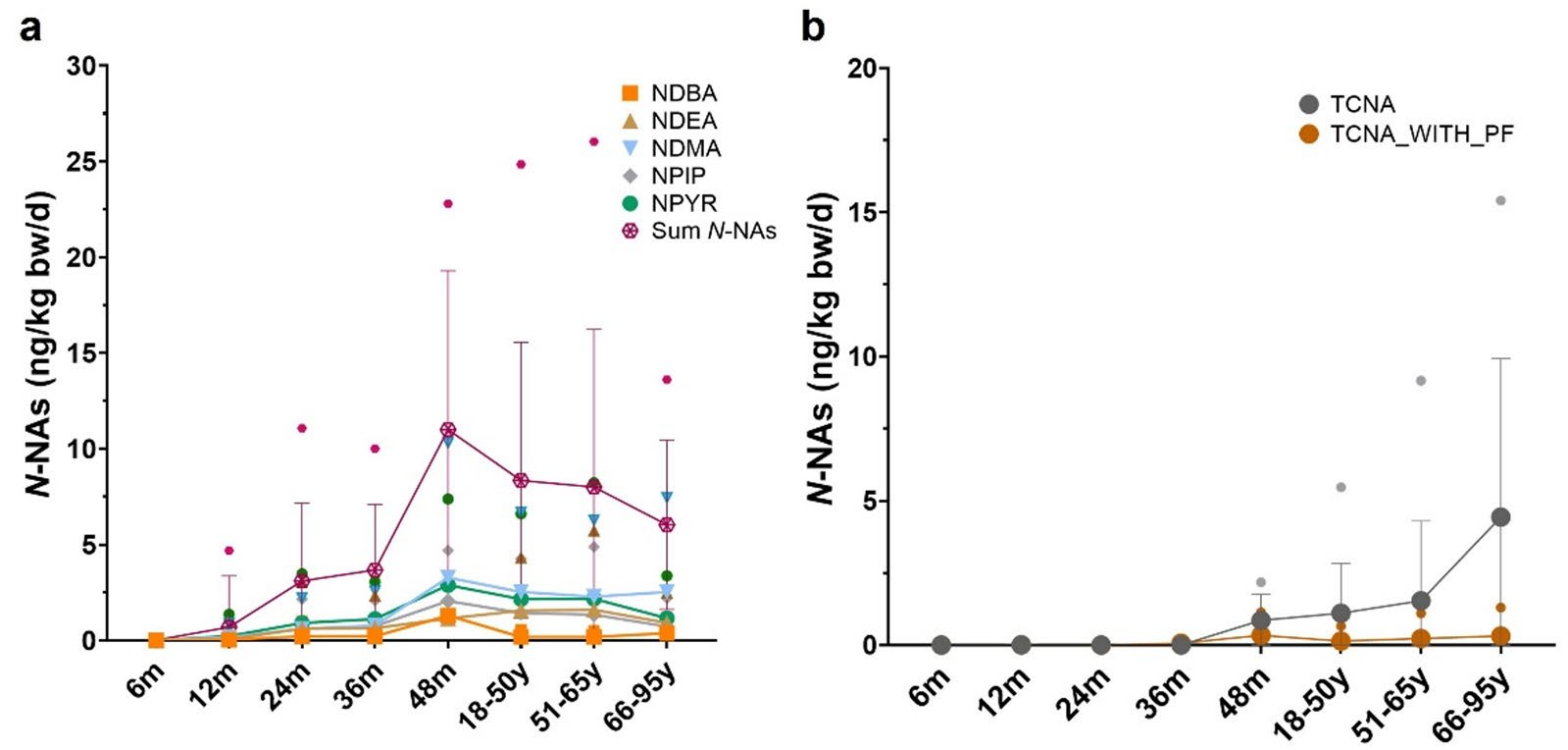
Dietary exposure to *N*-NAs across age groups. (**a**) Major *N*-NAs. (**b**) TCNA and TCNA with PF. Data is presented as mean ± SD and P95 (•). *N*-NAs, *N*-nitrosamines; NDBA, *N*-Nitrosodibutylamine; NDEA, *N*-Nitrosodiethylamine; NDMA, *N*-nitrosodimethylamine; NMEA, *N*-Nitrosomethylethylamine; NPIP, *N*-nitrosopiperidine; NPYR, *N*-nitrosopyrrolidine; TCNA, sum of 10 carcinogenic *N*-NAs; TCNA with PF, sum of 10 carcinogenic *N*-NAs considering potency factor.

The estimated intake of the main dietary *N*-NAs is shown in Fig. 1 and Supplementary Table S1. In children, individuals reached the greatest mean intake of the sum of *N*-NAs (11 ng/kg bw/d) at 48 months of age being NDMA, *N*-Nitrosopyrrolidine (NPYR), *N*-Nitrosopiperidine (NPIP) and *N*-Nitrosodibutylamine (NDBA) the main contributors (mean intakes of 3.28, 2.89, 2.06 and 1.30 ng/kg bw/d, respectively) (Fig. 1a). Subsequently, the mean total intake of *N*-NAs decreased progressively in adults (from 8 to 6 ng/kg bw/d) (Fig. 1a). In the case of the sum of the 10 carcinogenic *N*-NAs (TCNA), and the correction of this parameter by the potency factor (TCNA with PF) extracted from EFSA, the highest dietary exposure was observed in the 66–95 age group, with mean values of 4.44 and 0.32 ng/kg bw/d, and P95 values of 15 and 1 ng/kg bw/d, respectively (Fig. 1b).

**Table 3:** MOE values based on mean and P95 dietary exposure to *N*-NAs across age groups.

		6m *N* = 112	12m *N* = 109	24m *N* = 93	36m *N* = 84	48m *N* = 42	18-50y *N* = 21	51-65y *N* = 67	66-95y *N* = 94
NDMA	Mean	6,256,324	216,778	58,512	45,029	10,683	13,816	15,338	13,810
	P95	-	39,965	15,693	13,552	**3391**	**5225**	**5562**	**4695**
NPIP	Mean	29,901,520	394,600	102,008	83,403	30,121	43,366	46,281	85,048
	P95	-	65,180	28,719	29,765	13,178	14,555	12,709	28,214
NPYR	Mean	21,282,644	553,080	140,305	113,311	43,978	58,887	58,648	109,234
	P95	-	92,987	36,514	41,490	17,201	19,216	15,434	37,543
NDBA	Mean	60,790,699	1,600,035	301,774	288,197	47,789	348,872	339,231	162,246
	P95	-	-	62,633	82,485	21,054	110,503	122,838	52,295
NDEA	Mean	-	137,859	16,433	15,710	**8784**	**6429**	**6250**	10,856
	P95	-	-	**3290**	**4333**	**3008**	**2319**	**1750**	**4050**
NDPA	Mean	-	4,928,153	923,846	883,242	449,363	550,821	414,103	535,547
	P95	-	-	184,987	243,621	169,109	206,789	146,896	219,754
NMA	Mean	-	18,697,086	2,228,679	2,130,726	1,191,298	4,575,396	3,096,391	4,587,671
	P95	-	-	446,261	587,706	407,956	1,798,786	677,543	1,606,488
NMEA	Mean	-	-	-	-	-	105,828	132,855	249,246
	P95	-	-	-	-	-	31,804	33,060	51,171
NMOR	Mean	125,126,683	16,887,695	1,806,669	1,721,815	1,003,035	60,095	117,734	242,702
	P95	-	-	303,334	424,667	338,086	19,190	19,925	41,918
NSAR	Mean	-	7,011,409	835,755	799,023	446,737	2,004,944	1,923,075	1,842,774
	P95	-	-	167,349	220,390	152,984	688,047	479,687	653,790
Comb	Mean	-	-	-	-	-	1,176,003	232,548	211,467
	P95	-	-	-	-	-	-	52,551	43,745
Sum of *N*-NAs	Mean	666,667	14,599	**3224**	**2719**	**909**	**1198**	**1249**	**1656**
	P95	-	**2134**	**903**	**999**	**439**	**402**	**384**	**735**
TCNA	Mean	-	704,500	-	-	11,606	**8,992**	**6,489**	**2,251**
	P95	-	-	-	-	**4580**	**1827**	**1091**	**649**
TCNA with PF	Mean	-	1,967,786	-	134,095	29,792	69,948	42,953	30,842
	P95	-	-	-	-	**8757**	15,062	**9091**	**7722**

(-) not applicable. MOE values were calculated for each age group based on mean and P95 estimations of *N*-NAs dietary exposures, using the following BMDL10 experimental values (in mg/kg bw/d): NDMA (0.035), NDEA (0.010), NMOR (0.014), NPYR (0.127), NPIP (0.062), NDPA (0.062), NMOR (0.014), NDBA (0.062), NMA (0.062), NMEA (0.010), TCNA (0.010), TCNA with PF (0.010) and Comb. (0.010) and Sum of *N*-NAs (0.010)^1^. MOE values lower than 10,000 (in bold) may be considered a health concern (in bold). Comb., Combined nitroso compounds; MOE, margin of exposure; *N*-NAs, *N*-nitrosamines; NDBA, *N*-Nitrosodibutylamine; NDEA, *N*-Nitrosodiethylamine; NDMA, *N*-nitrosodimethylamine; NDPA, *N*-Nitrosodi-n-propylamine; NMA, *N*-nitrosomethylaniline; NMEA, *N*-Nitrosomethylethylamine; NMOR, *N*-Nitrosomorpholine; NPIP, *N*-nitrosopiperidine; NPYR, *N*-nitrosopyrrolidine; NSAR, *N*-Nitrososarcosine; TCNA, sum of 10 carcinogenic *N*-NAs; TCNA with PF, sum of 10 carcinogenic *N*-NAs considering potency factor

### Risk assessment of *N*-NA intake across age groups

The risk assessment of dietary *N*-NAs across age groups is presented in Table 3. According to MOE values, estimates lower than 10,000 were observed for all age groups, except for 6-month-old children. For the sum of *N*-NAs, the lowest MOE value based on mean dietary intake was noted for 48-month-old individuals among children (909), followed by adults from the 18–50 and the 51–65 age groups (1198 and 1249, respectively). Among all *N*-NAs, the lowest MOE estimates based on mean and P95 dietary exposure were observed for *N*-Nitrosodiethylamine (NDEA) in the 51–65 age group (MOE values of 6250 and 1750 based on mean and P95), followed by NDMA in the 48 month age group (MOE value of 3391 based on P95) as well as in the 66–95 age group, in the case of TCNA (MOE values of 2251 and 649 based on mean and P95) and TCNA with PF (MOE value of 7722 based on P95).

### Evolution of the dietary intake of precursors of *N*-NA formation across age groups: nitrates and nitrites

**Fig. 2:**
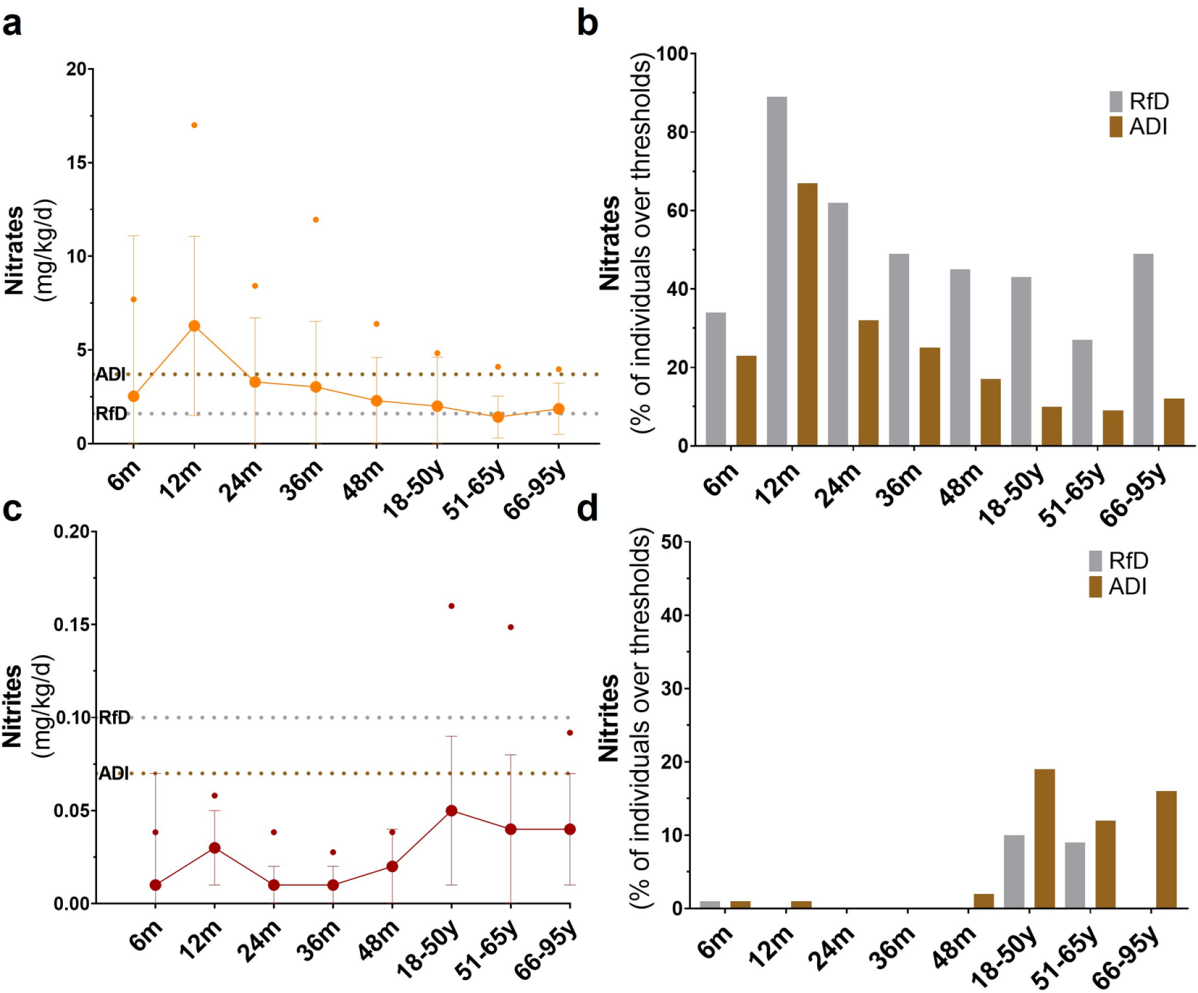
Dietary exposure to precursors of *N*-NA formation across age groups. (**a**) Nitrate consumption: data is presented as mean ± SD and P95 (•). (**b**) Percentage of individuals exceeding the RfD and ADI for nitrate. (**c**) Nitrite consumption: data is presented as mean ± SD and P95 (•). (**d**) Percentage of individuals exceeding the RfD and ADI for nitrite. ADI, acceptable daily intake; *N*-NAs, *N*-nitrosamines; RfD, reference dose.

Weight-standardized nitrate intake across all age groups is shown in Fig. 2 and Supplementary Table S1. Dietary nitrate was the highest in toddlers aged 12 months, with a mean intake of 6 mg/kg bw/d— two to four times higher than the ADI and RfD (Fig. 2a). At this age, 67 and 89% of individuals exceeded the ADI and RfD, respectively (Fig. 2b). In this age group, the P95 exposure peaked at 17 mg/kg bw/d and declined progressively to 4 mg/kg bw/d in individuals aged 66–95 years (Fig. 2a). Among adults, mean nitrate intake ranged from 1 to 2 mg/kg bw/d, with individuals in the 66–95 age group presenting the highest proportion consumers exceeding the RfD (49%) (Fig. 2b). The mean intake of nitrites was lower in children (0.01–0.03 mg/kg bw/d) compared to adults (0.04–0.05 mg/kg bw/d) (Fig. 2c). In adulthood, all age groups presented P95 exposures exceeding the ADI (0.09–0.16 mg/kg bw/d) (Fig. 2c), with 12–19% of volunteers surpassing this threshold (Fig. 2d).

### Evolution of the dietary intake of precursors and inhibitors of *N*-NA across age groups: ratios

**Fig. 3:**
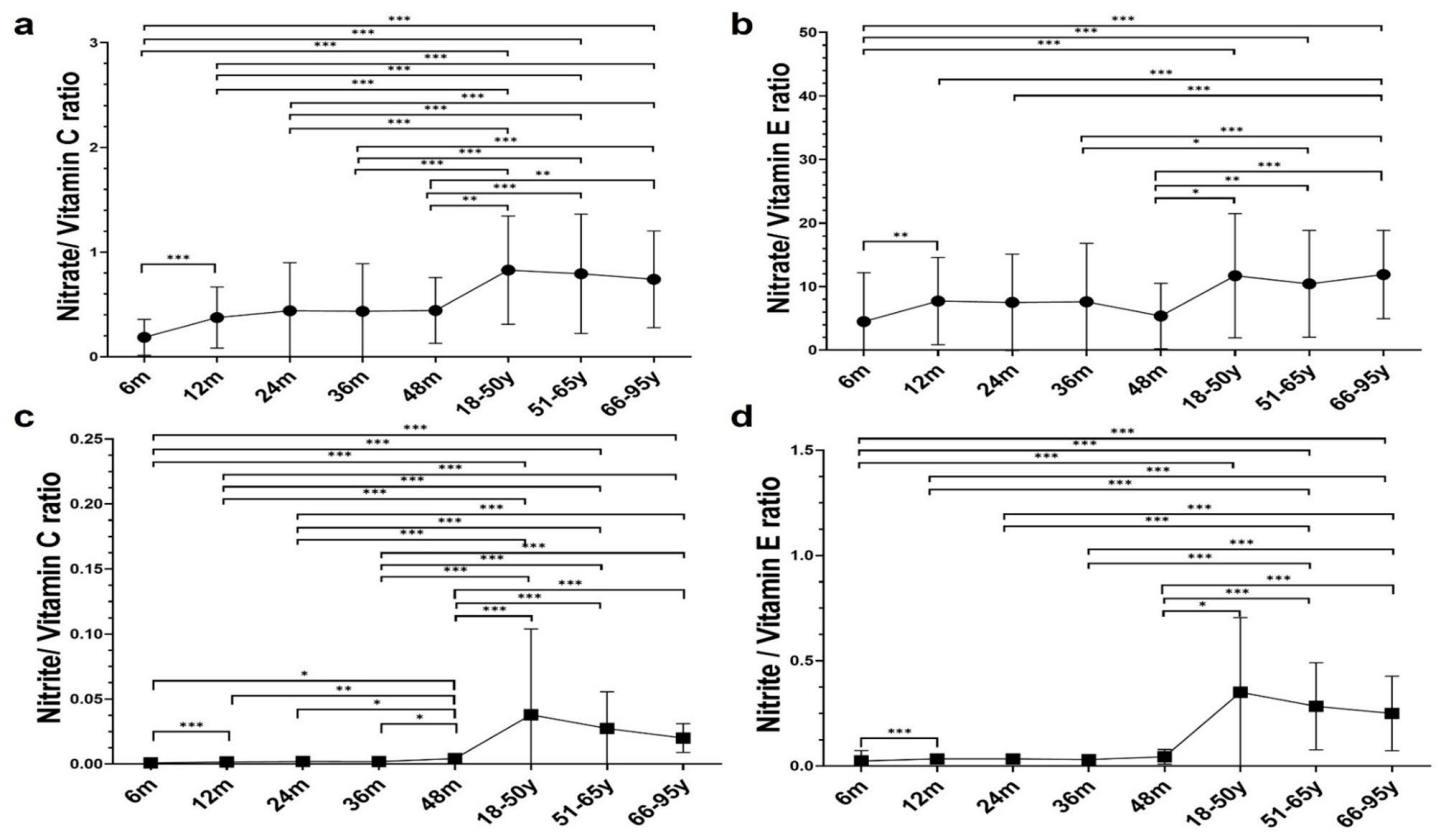
Dietary intake of inhibitor ratios of endogenous nitrosation across age groups. (**a**) Nitrate/Vitamin C. (**b**) Nitrate/Vitamin E. (**c**) Nitrite/Vitamin C. (**d**) Nitrite/Vitamin E. Data is presented as mean ± SD. Statistical significance was determined by Mann-Whitney U and Wilcoxon tests with Bonferroni correction (6 m *n* = 112; 12 m *n* = 108; 24 m *n* = 93; 36 m *n* = 84; 48 m *n* = 42; 18-50y *n* = 21; 51-65y *n* = 67; 66-95y *n* = 94). * *p-*value < 0.05, ** *p-*value < 0.01, *** *p-*value < 0.001.

Regarding other factors involved in endogenous nitrosation, a lower intake of nitrate/haem iron and nitrite/haem iron ratios was observed in children compared with adults (Supplementary Fig. S1). In the case of nitrate/vitamin C and nitrate/vitamin E ratios, an increasing trend was observed from childhood to adulthood (Fig. 3a, b). In children, a peak was noted at 12 months and it was maintained until 48 months of age. Compared with children, the ratio doubled in adults from the 18–50 year-age group and then remained similar in the 51–65 and 66–95 age groups. For nitrites, a similar positive trend was observed in the nitrite/vitamin C and nitrite/vitamin E ratios, with adults from the 18–50 age group exhibiting values eight to nine times higher than younger age groups and a decreasing trend occurred in individuals from the 66–95 group (Fig. 3c, d).

### Nitrate and nitrite as predictors of health risk factors

The potential of dietary intake of nitrate and nitrite over ADI and RfD thresholds to act as predictors of health-risk factors in adults is described in Table 4. In the 66–95 age group, consumption of nitrites over the ADI was associated with a reduced risk of being overweight or obese (*odds ratio* (OR): 0.129; 95% confidence interval (CI):0.033–0.510). Similarly, the consumption of nitrates over the RfD was associated with a reduced risk of obesity (OR: 0.157; 95% CI:0.045–0.539), high fasting glucose (OR: 0.225; 95% CI:0.066–0.760) and high triglycerides (OR: 0.121; 95% CI:0.017–0.873).

**Table 4:** Intake of nitrate and nitrite over the ADI and RfD as predictor of health-risk factors.

Age group	Health-risk factor	Intake	*N* (%)	Mean + SD (mg/kg bw/d)	OR (95% CI)	*p* - value
66-95y						
	Overweight or obesity					
		Nitrites < ADI	79 (84)	0.03 ± 0.02	-	
		Nitrites > ADI	15 (16)	0.08 ± 0.01	0.129 (0.033–0.510)	0.003
	Obesity					
		Nitrates < RfD	48 (51)	0.81 ± 0.43	-	
		Nitrates > RfD	46 (49)	2.97 ± 1.11	0.157 (0.045–0.539)	0.003
	High fasting glucose					
		Nitrates < RfD	48 (51)	0.81 ± 0.43	-	
		Nitrates > RfD	46 (49)	2.97 ± 1.11	0.225 (0.066–0.760)	0.016
	High triglycerides					
		Nitrates < RfD	48 (51)	0.81 ± 0.43	-	
		Nitrates > RfD	46 (49)	2.97 ± 1.11	0.121 (0.017–0.873)	0.036

Logistic regression models were performed for the following health-risk factors: overweight ( BMI 25.0–29.9 kg/m^2^), obesity (BMI ≥ 30.0 kg/m^2^); overweight or obesity (BMI ≥ 25.0 kg/m^2^); high fasting glucose (≥ 100 mg/dL); high cholesterol (≥ 200 mg/dL); high triglycerides (≥ 150 mg/dL); ethanol consumption; current smoking habit; practice of sport; previous diagnosis of hypertension, diabetes, asthma or allergies. For each variable, the intake below the ADI or RfD thresholds was considered the reference group. Only variables with statistically significant results are shown (*p-*value < 0.05). Values were adjusted for energy intake and gender. ADI; acceptable daily intake; RfD, reference dose; CI, confidence interval; OR, *odds ratio*.

**Fig. 4:**
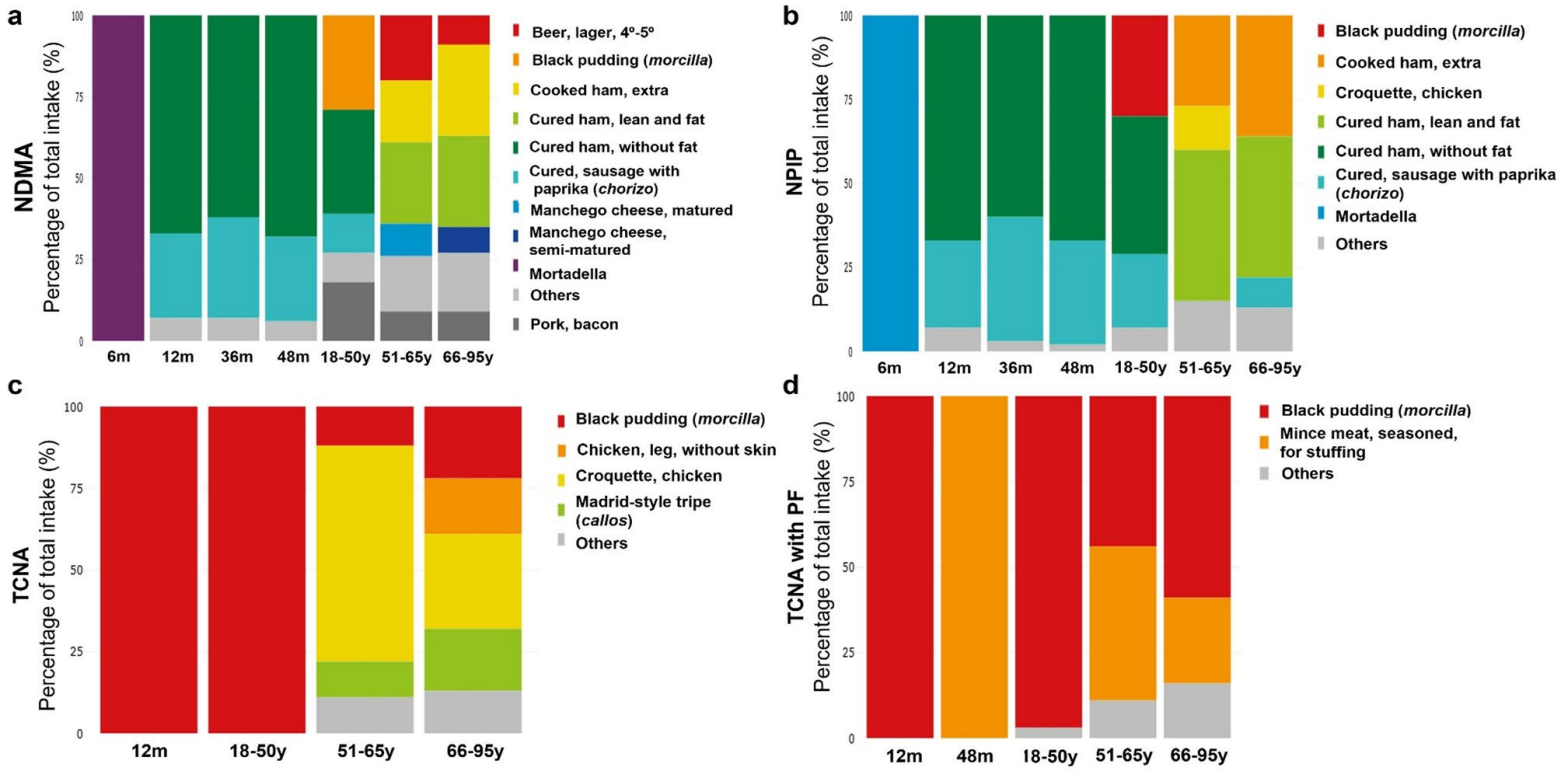
Dietary sources of *N*-NAs across age groups. (**a**) NDMA. (**b**) NPIP. (**c**) TCNA. (**d**) TCNA with PF. *N*-NAs, *N*- nitrosamines; NDMA, *N*- nitrosodimethylamine; NPIP, *N*- nitrosopiperidine; TCNA, sum of 10 carcinogenic *N*-NAs; TCNA with PF, sum of 10 carcinogenic *N*-NAs considering potency factor.

### Dietary sources of *N*-NAs, nitrates and nitrites across age groups

The intake of *N*-NAs is mostly derived from the consumption of meat and derivatives across all age groups (Fig. 4, Supplementary Fig. S2). The main contributors to the total intake of NDMA were processed meats, such as cured ham and cured sausage with paprika “*chorizo*”, from 12 month-old infants up to children aged 48 months. In adults, additional dietary sources including pork, bacon; cooked ham and beer, larger were noted (Fig. 4a). Similarly, black pudding “*morcilla”*, was one of the main contributors to the total intake of NDMA, NPIP, NPYR and NDBA in the 18–50 age group (Fig. 4a, b; Supplementary Fig. S2). Regarding TCNAs, meat derivatives were important contributors to the total intake in adults: croquette, chicken, in the case of TCNA; and minced meat, seasoned, for stuffing, in the case of TCNA with PF (Fig. 4c, d).

**Fig. 5:**
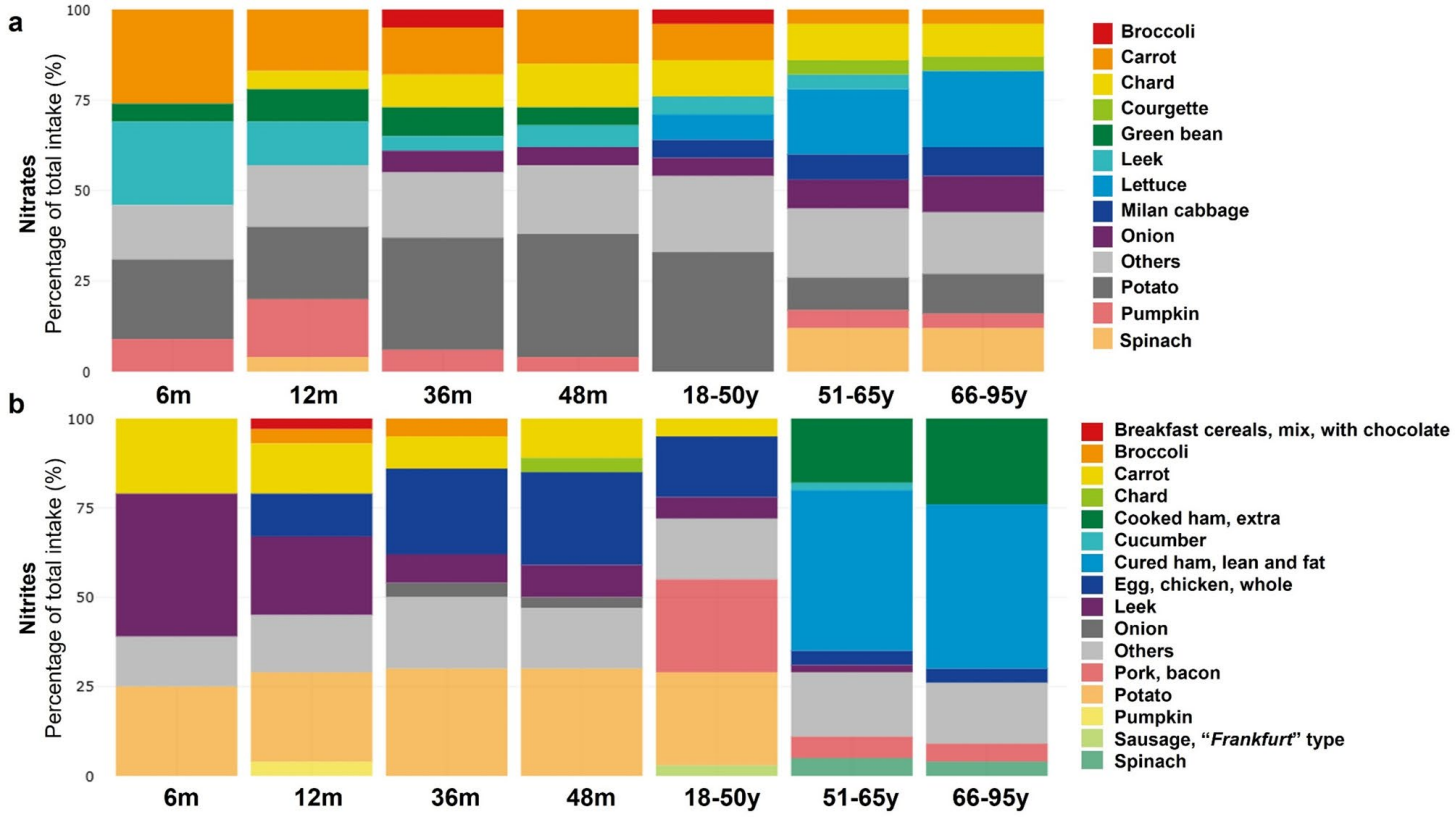
Dietary sources of precursors of *N*-NA formation across age groups. **a** Nitrates. **b** Nitrites.

The consumption of nitrates is mainly derived from plant-source foods (Fig. 5a, Supplementary Fig. S3). From 6 to 48 months of age, their main contributors were carrots, potatoes, leek and chard whereas lettuce, onion, cabbage and spinach were additional important contributors in adults (from 18 to 50 to 66–95 age groups). In the case of nitrites, the percentage of the total intake derived from animal sources increased progressively in childhood from 12 month-old infants (8%) up to 48 month toddlers (52%) and 18–95 year-old adults (85–90%) (Supplementary Fig. S3). Specifically, across age groups, a reduced contribution of leek to the total intake was observed, in parallel with a greater contribution of eggs in children; and pork, bacon; cooked ham and cured ham, in adults (Fig. 5b).

## Discussion

The dietary assessment of *N*-NAs has gained increasing attention due to their potential health risks^1^. Classified as probably carcinogenic to humas by the IARC^2^, *N*-NAs intake has been linked to a higher risk of gastrointestinal cancers and other malignancies^1^. Moreover, recent findings have demonstrated a positive association between dietary *N*-NAs intake and faecal concentrations of NOCs, in parallel with greater colonic mucosal damage^32^, underscoring the importance of evaluating dietary exposure to *N*-NAs. To the best of our knowledge, no studies have comprehensively evaluated dietary exposure and main sources of *N*-NAs and their precursors, nitrates and nitrites, across all age groups. In this work, we assessed weight-standardized dietary exposure to *N*-NAs, nitrates and nitrites and evaluated the dietary potential for endogenous nitrosation across different age groups in a sample of individuals from Asturias (northern Spain) ranging from 6-month-old to 95-year-old participants.

The dietary exposure to the sum of *N*-NAs assessed in this work (0–11 ng/kg bw/d) lies within the range of the mean levels described by EFSA (0–12 ng/kg bw/d)^1^ and previous Spanish studies^13^, with values approximately 3 orders of magnitude lower than the conservative BMDL10 threshold (10 µg/kg bw/d)^1^. The intake of *N*-NAs in infants was first quantified at 6 months of age during the weaning period, when 1% of the total sample consumed five *N*-NAs derived from mortadella. Subsequently, *N*-NA consumption increased progressively with the acquisition of the family diet in children, with the highest mean intake observed at 48 months (93–98% consuming the same *N*-NAs). Notably, NDMA exhibited the highest contribution to total exposure (P95 0-10.32 ng/kg bw/d), closely aligning with estimates reported by EFSA for infants and children (P95 0-9.8 ng/kg bw/d). However, this intake translates to an absolute exposure of 186 ng/d at 48 months of age (based on a mean bw of 18.02 kg), exceeding the established acceptable intake of 96 ng/d^1^. Furthermore, the mean consumption of processed meats in 48-month-old individuals surpassed the 25 g/d threshold associated with an increased risk of colorectal cancer^33^. Considering that this relatively high consumption of processed meat persists in adults (up to 62 g/d), and that mortadella, cured ham and cured sausage with paprika “*chorizo”* were the main contributors to *N*-NAs intake which is consistent with previous findings among Spanish individuals^13^, we propose that 48 months of age should be considered a critical period due to the elevated risk of exceeding acceptable levels of exposure. Although evidence on the effects of elevated dietary exposure to *N*-NAs during early life stages remains limited, children may exhibit greater susceptibility to carcinogenic effects than adults^34^.

The MOE estimates obtained in this study are consistent with those reported by EFSA^1^ in both children and adults. In the present work, MOE values based on mean and P95 dietary exposure below 10,000 were observed for NDEA and NDMA—which, according to established risk assessment criteria for genotoxic and carcinogenic substances^31^, indicate a potential health concern, particularly during early childhood, since 48-month-old children exhibited the lowest MOE values for *N*-NAs. Efforts to reduce processed meat consumption among young children are warranted, given its high content of total and saturated fats—up to 90 and 25%, respectively, in meat derivatives such as bacon^35^ —and the well-established associations with adverse health outcomes, including type 2 diabetes, cardiovascular disease, and all-cause mortality^36^. Therefore, the findings of this study highlight 48 months of age as a critical window for dietary intervention, during which the transition to the family diet should be carefully managed to minimize exposure to potential carcinogenic compounds.

On the other hand, nitrates and nitrites are well-known dietary precursors of endogenous nitrosation^12^ and recent evidence highlights a source-specific effect^37,38^. In this work, the mean consumption of nitrates from infants to children (up to 6.3 mg/kg bw/d) and adults (up to 2.0 mg/kg bw/d) aligns with the results reported by EFSA (up to 4.6 mg/kg bw/d in infants and toddlers and up to 2.5 mg/kg bw/d in adults)^27^. Indeed, although EFSA reported P95 dietary nitrate exposures up to two to three times the ADI^27^, this study identified even higher exceedances—up to fivefold in 12-month-old toddlers— suggesting a potential overexposure to this compound at early stages. However, the latest EFSA assessment emphasized the importance of distinguishing between animal- and plant-derived nitrate sources^27^, as plant-based nitrates have been associated with cardiovascular benefits^39,40^ and a higher risk of all-cause mortality was noted for higher intakes of animal-source nitrate^37,38^. In the present study, 99–100% of nitrate intake across age groups originated from plant sources, a markedly higher proportion than the 45% reported in Danish adults^17^, likely reflecting the lower red meat consumption among Spanish adults (47 vs. 81 g/d). Thus, despite exceeding threshold levels, the predominance of vegetable-derived nitrates suggests a lower nitrosation potential and reduced associated health risk, which aligns with the reduced risk of obesity, high circulating fasting glucose, and high triglycerides observed among older adults with higher nitrate consumption.

In the case of nitrites, 12–19% of adults exceeded the more conservative health-based guidance threshold and the mean intake levels (0.04–0.05 mg/kg bw/d) were consistent with EFSA estimations for this age group (0.03–0.05 mg/kg bw/d)^29^. In contrast, infants and children exhibited lower intake values (0.01–0.03 mg/kg bw/d) compared to EFSA data (0.05–0.15 mg/kg bw/d), with only 1% of infants aged 6 and 12 months surpassing the ADI, indicating limited exposure risk to nitrites in early life. In younger age groups animal-derived nitrite accounted for 8–52% of total intake, with eggs emerging as an important contributor whereas animal sources accounted for 90% of total nitrite intake in adults, higher than the 36% reported for healthy Danish adults^17^. This difference may be reflected by the higher consumption of processed meats among Spanish adults (61 vs. 22 g/d), with cooked and cured ham identified as the principal contributors. Although animal-derived nitrite intake has been positively associated with increased risks of cardiovascular disease, cancer-related mortality, or obesity^17,37^ in the present study, those elderly individuals with a higher consumption of nitrites were associated with a reduced risk of being overweight or obese, probably due to the decreasing tendency of nitrosation potential observed in later stages of life. However, considering that lifetime cumulative dose of carcinogenic compounds may increase carcinogenic potency in older adults^25^, further studies focusing on this age group are warranted to better elucidate these associations.

In the complex gastrointestinal environment, the presence of co-occurring dietary components after the consumption of a wide variety of foods may influence the potential of endogenous nitrosation^17^. For example, the formation of *N*-NAs from nitrate, after its reduction to nitrite, may be augmented in the presence of meats due to their content of haem iron, and an increased risk of colorectal cancer has been observed in individuals presenting a higher consumption of nitrates and red meat^16^. In the present study, an increasing intake of nitrate and nitrite to haem iron ratios was noted from childhood to adulthood, suggesting a possible greater nitrosation potential in adults. In this line, although previous studies have reported a positive association between dietary haem iron and faecal NOCs, indicating a potential promotion of endogenous nitrosation^32^, further research is needed to clarify these relationships. On the other hand, the formation of *N*-NAs may be inhibited in the presence of vitamins^15^, and previous authors have shown that a higher consumption of nitrate is associated with an increased risk of colorectal cancer when consumed in parallel with a low dietary intake of vitamin C^16^. In this work, a higher intake of both nitrate and nitrite to vitamin C and E ratios was noted in adults, especially in the 18–50 age group compared to toddlers and younger individuals, suggesting a potential promotion of the endogenous nitrosation process in older groups.

To our knowledge, this is one of the first studies in Spain to analyse source-specific dietary intake of *N*-NAs, nitrates, and nitrites across a broad age range—from infants and toddlers to children, and younger, middle-aged, and elderly adults. Although extrapolation of the results may be limited due to the geographical and demographic characteristics of the participants (Asturias, northern Spain), previous studies within middle-aged adults that included this and other southern regions reported similar values of consumption of NDMA^13^ and main *N-*NA dietary sources (e.g. cooked ham, “*chorizo*” or “*morcilla”*)^41^. The absence of data in adolescents is a limitation of this study although similar *N*-NA intake estimates for adolescents and children were reported by EFSA^1^. In contrast to previous studies relying on 24-hour dietary recalls, the present one utilized a previously validated Food Frequency Questionnaire (FFQ) for nitrates, nitrites and *N*-NAs, allowing for a more robust characterization of habitual dietary intake patterns^42^. Although reliance on secondary data is a common limitation of nutritional studies, the use of a harmonized database mainly compiled from European sources for a wide range of food (348 items), allows comparison with results published by reference institutions, including EFSA or EPIC. Additionally, although nitrates and nitrites have been detected in human breast milk—approximately 0.19 and 0.08 mg/100 mL, respectively^43^ —the contribution of human breast milk to total intake of nitrates and nitrites was not considered in the present analysis due to numerous confounding factors influencing their nitrate and nitrite concentrations, including gestational age at birth, milk type (colostrum, transitional, or mature), thermal processing, storage conditions, and the composition of the infant gut microbiota^15,44^. Moreover, it has been proposed that conventional threshold values, such as the ADI, may not be appropriate for application during early developmental stages.

The risk assessment performed in this work revealed that the dietary intake of most common *N*-NAs, mainly derived from processed meats, may pose health concerns across all age groups. Therefore, efforts should focus on minimizing their formation in foods and their dietary intake—particularly from nitrite-containing, animal-derived products—among vulnerable populations. This study highlights early childhood as a critical window for intervention to minimize exposure to potential carcinogenic substances, supporting the development of public health policies aimed at mitigating associated health risks.

## Methods

### Study sample

This is an observational and descriptive study in which *N*-NAs intake was calculated from 671 dietary questionnaires belonging to 116 children followed up longitudinally from 6 months after birth to 4 years at four sampling points: 6, 12, 36 and 48 months (54–64% males) and of 182 adults aged 19 to 95 years (33–36% male). All volunteers were recruited in Asturias region (North of Spain) in the context of previous projects of the research group: PCIN-2015-233 (Project EarlyMicroHealth from the EU Joint Program Initiative HDHL) (MINECO/FEDER, UE); 2017/00001/003/005/0 (Project Diet, Rhythms and Early Acquisition Microbiota (DREAMS)); e-CENIT Project SENIFOOD financed by the Spanish Ministry of Science and Innovation and Biopolis SL; and Project “Estrategias Dietéticas para la modulación de la Microbiota y la promoción de un envejecimiento Saludable”, funded by Alimerka Foundation (Llanera, Spain). Ethical approval was obtained from the Bioethical Committee of CSIC and from the Regional Ethics Committee for Clinical Research of the Principality of Asturias. All protocols were performed in line with the principles stated in the Declaration of Helsinki, the Bioethics Convention of University of Oviedo, the Council of Europe’s Convention on Human Rights and Biomedicine and in Spanish legislation on bioethics. All the volunteers, or their legal representatives, gave written informed consent. The inclusion criteria considered for this study were: for children, being born healthy without any condition that would affect normal intake pattern or the administration of drugs or special care; and for adults, not having been diagnosed with a major health condition (cancer, Parkinson’s disease, irritable bowel disease, or autoimmune diseases) and not having consumed antibiotics or probiotics during the month previous to recruitment. All measurements were carried out in accordance with approved guidelines and regulations.

### General characteristics and anthropometrical parameters

Gender and age were registered for all individuals. In adults, information about lifestyle factors such as alcohol consumption, smoking habit (current smoker, former smoker or never smoker), physical activity and presence of previous pathologies (hypertension, diabetes, asthma or allergies) was registered. For each volunteer, height (m) and weight (kg) were measured by standard methods using calibrated and suitable equipment: a stadiometer with an accuracy of ± 1 mm (Año-Sayol, Barcelona, Spain) and a scale with an accuracy of ± 100 g (Seca, Hamburg, Germany). For children, the body mass index (BMI) Z-score was obtained relative to WHO Child Growth Standards^45^, using WHO ANTHRO, Software for Calculating Anthropometry, version 3^46^. BMI Z-score in children was classified as underweight (from-5.99 to-1.00), normal weight (from-0.99 to 0.99), overweight (from 1.00 to 1.99), and obese (from 2.00 to 2.99). For adults, BMI was calculated using the formula: weight (kg)/height (m)^2^ and categorized according to the Spanish Society for the Study of Obesity (SEEDO) criteria as underweight (< 18.5 kg/m^2^), normal weight (18.5–24.9 kg/m^2^), overweight (25.0–29.9 kg/m^2^), and obese (≥ 30.0 kg/m^2^)^47^.

### Blood analyses

In adults, fasting blood samples were drawn by venipuncture and collected in separate tubes for serum and plasma. Blood samples were kept on ice and centrifuged (1000 x g, 15 min) within 2–4 h after collection and aliquots of serum and plasma were stored at − 20 °C until analysis were performed. Analysis of fasting plasma glucose, cholesterol and triglycerides were performed by using an automated biochemical autoanalyzer. According to previous authors, high fasting glucose, high triglycerides and high cholesterol were defined as serum concentrations over 100, 150 and 200 mg/dL, respectively^48–50^.

### Dietary assessment

Dietary information was registered from each participant or their legal representative through a FFQ, previously validated for the quantification of *N*-NAs, nitrates and nitrites^51^. In addition to food consumption, cooking methods (boiled, fried, grilled, baked/broiled, or barbecued), food preferences (undercooked, medium, well-done, very well-done), as well as information on skin consumption and/or cooking were recorded, as previously described^51,52^. To facilitate the completion of the information, the validated questionnaire contained 31 photographs specifically developed for this project, showing foods cooked to progressively higher degree of doneness. Portion size was standardized using a validated photograph album adapted from the Pilot Study for Assessment of Nutrient intake and Food Consumption Among Kids in Europe (PANCAKE) study^53^.

Food consumption was classified into food groups according to the Centre for Higher Education in Nutrition and Dietetics (CESNID). These databases were used to transform food consumption into energy intake^54^. The food content of nitrates, nitrites and the following *N*-NAs (per each 100 g) was compiled: NDMA, NPIP, NPYR, NDBA, NDEA, *N*-Nitrosodi-n-propylamine (NDPA), *N*-Nitrosomethylaniline (NMA), *N*-Nitrosomethylethylamine (NMEA), *N*-Nitrosomorpholine (NMOR), *N*-Nitrososarcosine (NSAR) and different combinations of NOCs (Comb.). According to EFSA CONTAM Panel, TCNA and the corrected parameter (TCNA with PF), were used to account for differences in the carcinogenic potency between different *N*-NAs^1^. Data for 348 items were obtained from the European Prospective Investigation into Cancer and Nutrition (EPIC) Potential Carcinogen Database^55^, the EFSA database^1,56^, and previous works^3,8,57,58^. When multiple sources were available for the same item, weighted averages were calculated based on the number of studies contributing to each determination. The harmonized dataset is available in Supplementary Material 2. The intake of potential carcinogenic compounds was weight-standardized, and the sum of *N*-NAs was calculated in this work as: NDMA + NDEA + NPYR + NPIP + NDPA + NMOR + NDBA + NSAR + NMA + NMEA + Comb.

The dietary risk characterization of *N*-NAs intake was performed using the MOE, which quantifies the level of concern associated with dietary exposure to a genotoxic and carcinogenic compound^30^. MOE was calculated using the formula: BMDL10 (mg/kg bw/d) / estimated dietary exposure (mg/kg bw/d), as described in previous works^59^. The following BMDL10 values (mg/kg bw/d) were used as reference points for each *N*-NA: NDMA (0.035), NDEA (0.010), NMOR (0.014), NPYR (0.127), NPIP (0.062), NDPA (0.062), NMOR (0.014), NDBA (0.062), NMA (0.062), NMEA (0.010), TCNA (0.010), TCNA with PF (0.010), Comb. (0.010) and Sum of *N*-NAs (0.010)^1^. BMDL10 values 10,000 times higher than the exposure (i.e., MOE ≥ 10,000) for genotoxic chemicals is considered of low concern from a public health perspective^60^.

The toxicological reference values ADI and RfD were used for the assessment of dietary nitrates (3.7 and 1.6 mg/kg bw/d) and nitrites (0.07 and 0.01 mg/kg bw/d), respectively^27–29^. These values represent the estimated safe daily intake over a lifetime without appreciable health risk^61^. Based on the potential of dietary components to inhibit (vitamin C and E) or promote (haem iron) endogenous nitrosation^16^, the endogenous nitrosation potential of dietary intake was calculated using the following ratios: nitrate/vitamin C, nitrate/vitamin E, nitrate/ haem iron and nitrite/vitamin C, nitrite/vitamin E, nitrite/haem iron. Haem iron was calculated as the sum of iron derived from animal sources, including meat and meat products, milk and dairy products, eggs, fish and seafood^62^.

The dietary sources of the analysed compounds were identified as food items cumulatively contributing to at least 80% of total intake in each case. To facilitate this analysis, the FoodSources Shiny-based application was developed specifically for this project. The open-access tool is available through any web browser at https://zapicoaida.shinyapps.io/foodsources/. Users can upload input data as spreadsheet (XLSX) or comma-separated text (CSV) formats. Subsequently, the dietary compound of interest is selected, summary tables and plots are displayed interactively, while the data analysed can be downloaded as XLSX and/or PNG files. Analyses can be performed for the total sample or stratified by user-defined categories (e.g., age groups). The functionality of FoodSources can be explored using the example data (Supplementary Material 3), where each Excel sheet (dietary data and classification) represents an individual file that should be uploaded separately.

### Statistical analysis

Data were analysed using the IBM SPSS software version 25.0 (IBM SPSS, Inc., Chicago, IL, USA). Goodness of fit to the normal distribution was checked by means of the Kolmogorov–Smirnov test. As normality of the variables was not achieved, nonparametric tests were used. Categorical variables were summarized as percentages and continuous variables as mean and standard deviations. In the case of nitrates, nitrites and *N*-NAs, P95 were also reported. For continuous variables, Wilcoxon test was applied in children to assess changes across different ages within the same individuals (sampling points at 6, 12, 36, and 48 months) and Mann-Whitney U test was used for independent comparisons between each sampling point in children and each adult age group (18–50, 51–65 and 66–95 years), as well as for pairwise comparisons among the adult age groups. A Bonferroni correction was applied to adjust for multiple comparisons. The FoodSource Shiny based application was developed in RStudio (version 1.4.1103), using the following packages: ggplot2, dplyr, tidyr, plotly, readxl and writexl^63–68^. Logistic regression models were conducted to evaluate whether nitrate and nitrite intakes exceeding the RfD and ADI thresholds were predictive of the following health-risk outcomes: overweight; obesity; overweight or obesity (combined); high fasting glucose; high circulating cholesterol; high circulating triglycerides; ethanol consumption; current smoking habit; practice of sport; previous diagnosis of hypertension, diabetes, asthma or allergies. All models were adjusted for energy intake and gender. Graphical representations were performed using GraphPad Prism version 8.0.2 (GraphPad Software, San Diego, CA, USA).

## Supplementary Information

Below is the link to the electronic supplementary material.

Supplementary Material 2

Supplementary Material 3

Supplementary Material 1

## Data Availability

The datasets generated during and/or analysed during the current study are not publicly available due to ethical reasons but are available from the corresponding author on reasonable request.

## Abbreviations

ADI: Acceptable daily intake
BMDL10: Benchmark Dose Lower Confidence Limit 10%
BMI: Body mass index
CESNID: Centre for Higher Education in Nutrition and Dietetics
CI: Confidence Interval
Comb.: Combined NOCs
EFSA: European Food Safety Authority
EPIC: European Prospective Investigation into Cancer and Nutrition
FFQ: Food Frequency Questionnaire
IARC: International Agency for Research on Cancer
Kg bw: Kilogram of body weight
MOE: Margin of exposure
*N*-NAs: *N*-Nitrosamines
NDBA: *N*- Nitrosodibutylamine
NDEA: *N*- Nitrosodiethylamine
NDMA: *N*- Nitrosodimethylamine
NDPA: *N*- Nitrosodi-n-propylamine
NMA: *N*- Nitrosomethylaniline
NMEA: *N*- Nitrosomethylethylamine
NMOR: *N*- Nitrosomorpholine
NOCs: *N*- Nitroso compounds
NPIP: *N*- Nitrosopiperidine
NPYR: *N*- Nitrosopyrrolidine
NSAR: *N*- Nitrososarcosine
OR: *Odds ratio*
P95: 95th percentile
PANCAKE: Pilot Study for Assessment of Nutrient intake and Food Consumption Among Kids in Europe
RfD: reference dose
SEEDO: Spanish Society for the Study of Obesity
TAC: Total antioxidant capacity
TCNA: The sum of 10 carcinogenic *N*-NAs
TCNA PF: The sum of 10 carcinogenic *N*-NAs with potency factor
