# Supplementary material for: Dietary exposure to N-nitrosamines and their precursors: an age-stratified assessment
Source: Sci Rep. 2026 Jan 19;16:4047. doi: 10.1038/s41598-025-34144-7 (PMC12855995; doi:10.1038/s41598-025-34144-7)
Supplement: Supplementary file 1 — Supplementary Material 2 [file 41598_2025_34144_MOESM1_ESM.pdf]

SUPPLEMENTARY INFORMATION

Title

Dietary Exposure to *N*-Nitrosamines and their Precursors: An Age-Stratified Assessment

Authors

Aida Zapico[1,2]*, David Herrero-Morin[3], Silvia Arboleya[2,4], Clara G. de los Reyes-Gavilán[2,4], Miguel Gueimonde[2,4],Sonia González[1,2]*

[1]Department of Functional Biology, University of Oviedo, 33006 Oviedo, Spain.

[2]Diet, Microbiota and Health Group, Instituto de Investigación Sanitaria del Principado de Asturias (ISPA), 33011 Oviedo, Spain.

[3]Pediatrics Service, Centro Atención Primaria Infiesto, SESPA, 33530 Piloña, Spain.

[4]Department of Microbiology and Biochemistry of Dairy Products, Instituto de Productos Lácteos de Asturias (IPLA-CSIC), 33011 Oviedo, Spain.

Corresponding author

* zapicoaida@uniovi.es (A.Z.) and soniagsolares@uniovi.es (S.G.)

Supplementary Table S1. Intake of nitrates, nitrites and *N*-NAs in the sample study across age.

(ng/kg bw/d)	6m N=112	12m N=109	24m N=93	36m N=84	48m N=42	18-50y N=21	51-65y N=67	66-95y N=94
Nitrates (mg/kg bw/d)	2.53 ± 8.57 (7.70)a,i,j,k,l	6.29 ± 4.78 (17.00)bb	3.29 ± 3.43 (8.42)c,m,n,o	3.03 ± 3.49 (11.95)d,k,n,p,q,r,s	2.29 ± 2.31 (6.39)e,l,o,r,t,u,v	2.00 ± 2.62 (4.83)f,i,s,v,w,x	1.42 ± 1.12 (4.10)g,j,p,t,w,y	1.86 ± 1.37 (3.97)h,m,q,u,x, y
Nitrites (mg/kg bw/d)	0.01 ± 0.06 (0.04)a,i,j	0.03 ± 0.02 (0.06)bb,k,l,m,n	0.01 ± 0.01 (0.04)c,i,o,p,q	0.01 ± 0.01 (0.03)d,j,o,r	0.02 ± 0.02 (0.04)e,m,p,r	0.05 ± 0.04 (0.16)f,n,q,s,t	0.04 ± 0.04 (0.15)g,k,s,u	0.04 ± 0.03 (0.09)h,l,t,u
NDMA	0.01 ± 0.06 (0.00)a,i	0.16 ± 0.70 (0.88)b,i	0.60 ± 0.83 (2.23)c,j	0.78 ± 0.80 (2.58)d,j	3.28 ± 2.81 (10.32)e,k,l,m	2.53 ± 1.91 (6.70)f,m,n,o	2.28 ± 2.15 (6.29)g,k,n,p	2.53 ± 2.21 (7.45)h,l,o,p
NPIP	0.00 ± 0.02 (0.00)a,i	0.16 ± 0.67 (0.95)b,i	0.61 ± 0.82 (2.16)c,j,k	0.74 ± 0.73 (2.08)d,k,l,m,n	2.06 ± 1.75 (4.70)e,o	1.43 ± 1.34 (4.26)f,n,o,p,q	1.34 ± 1.48 (4.88)g,l,p	0.73 ± 0.70 (2.20)h,j,m,q
NPYR	0.01 ± 0.06 (0.00)a,i	0.23 ± 1.03 (1.37)b,i	0.91 ± 1.26 (3.48)c,j	1.12 ± 1.14 (3.06)d,j,k,l	2.89 ± 2.54 (7.38)e,m,n	2.16 ± 2.24 (6.61)f,l,n,o,p	2.17 ± 2.50 (8.23)g,m,o	1.16 ± 1.15 (3.38)h,k,p
NDBA	0.00 ± 0.01 (0.00)a,i	0.04 ± 0.24 (0.00)b,i	0.21 ± 0.41 (0.99)c,j	0.22 ± 0.30 (0.75)d,j,k,l	1.30 ± 1.13 (2.94)e	0.18 ± 0.21 (0.56)f,l,m,n	0.18 ± 0.19 (0.50)g,k,m	0.38 ± 0.39 (1.19)h,n
NDEA	0.00 ± 0.00 (0.00)a,i	0.07 ± 0.42 (0.00)b,i	0.61 ± 1.23 (3.04)c,j	0.64 ± 0.89 (2.31)d,j,k	1.14 ± 0.95 (3.32)e,k,l,m,n	1.56 ± 1.50 (4.31)f,n,o,p	1.60 ± 1.72 (5.71)g,l,o	0.92 ± 0.85 (2.47)h,m,p
NDPA	0.00 ± 0.00 (0.00)a,i	0.01 ± 0.08 (0.00)b,i	0.07 ± 0.14 (0.34)c,j	0.07 ± 0.10 (0.25)d,j,k,l	0.14 ± 0.11 (0.37)e,k,m,n,o	0.11 ± 0.11 (0.30)f,l,o,p,q	0.15 ± 0.16 (0.42)g,m,p,r	0.12 ± 0.11 (0.28)h,n,q,r
NMA	0.00 ± 0.00 (0.00)a,i	0.00 ± 0.02 (0.00)b,i	0.03 ± 0.06 (0.14)c,j	0.03 ± 0.04 (0.11)d,j,k,l,m,n	0.05 ± 0.04 (0.15)e,m	0.01 ± 0.01 (0.03)f,n,o,p	0.02 ± 0.04 (0.09)g,k,o,q	0.01 ± 0.01 (0.04)h,l,p,q
NMEA	0.00 ± 0.00 (0.00)a,i,j,k,l	0.00 ± 0.00 (0.00)b,i,m,n,o	0.00 ± 0.00 (0.00)c,j,m,p,q	0.00 ± 0.00 (0.00)d,k,n,p,r	0.00 ± 0.00 (0.00)e,l,o,q,r	0.09 ± 0.13 (0.31)f,s,t	0.08 ± 0.09 (0.30)g,s	0.04 ± 0.05 (0.20)h,t
NMOR	0.00 ± 0.00 (0.00)a,i	0.00 ± 0.00 (0.00)b,i	0.01 ± 0.02 (0.05)c,j,k	0.01 ± 0.01 (0.03)d,j,l,m	0.01 ± 0.01 (0.04)e,k,m,n,o	0.23 ± 0.38 (0.73)f,p	0.12 ± 0.25 (0.70)g,n,p,q	0.06 ± 0.20 (0.33)h,l,o,q
NSAR	0.00 ± 0.00 (0.00)a,i	0.01 ± 0.05 (0.00)b,i	0.07 ± 0.15 (0.37)c,j	0.08 ± 0.11 (0.28)d,j,k,l,m,n	0.14 ± 0.12 (0.41)e,m	0.03 ± 0.03 (0.09)f,n,o,p	0.03 ± 0.04 (0.13)g,k,o,q	0.03 ± 0.03 (0.09)h,l,p,q
Comb	0.00 ± 0.00 (0.00)a,i,j,k,l,m	0.00 ± 0.00 (0.00)b,j,n,o,p,q	0.00 ± 0.00 (0.00)c,k,n,r,s,t	0.00 ± 0.00 (0.00)d,l,o,r,u,v	0.00 ± 0.00 (0.00)e,m,p,s,u,w,x	0.01 ± 0.04 (0.00)f,i,q,t,v,x,y	0.04 ± 0.20 (0.19)g,w,y	0.05 ± 0.09 (0.23)h
Sum of *N*-NAs	0.02 ± 0.16 (0.00)a,i	0.69 ± 2.68 (4.69)b,i	3.10 ± 4.08 (11.07)c,j	3.68 ± 3.42 (10.01)d,j	11.00 ± 8.29 (22.79)e,k,l	8.35 ± 7.20 (24.84)f,l,m,n	8.01 ± 8.22 (26.03)g,k,m,o	6.04 ± 4.42 (13.61)h,n,o
TCNA	0.00 ± 0.00 (0.00)a,i,j,k	0.01 ± 0.15 (0.00)b,i,l,m	0.00 ± 0.00 (0.00)c,j,l,n	0.00 ± 0.00 (0.00)d,k,m,n	0.86 ± 0.90 (2.18)e,o,p,q	1.11 ± 1.72 (5.47)f,q,r,s	1.54 ± 2.76 (9.17)g,o,r,t	4.44 ± 5.49 (15.41)h,p,s,t
TCNA with PF	0.00 ± 0.00 (0.00)a,i,j,k	0.01 ± 0.05 (0.00)b,i,l,m	0.00 ± 0.00 (0.00)c,j,l,n	0.07 ± 0.48 (0.00)d,k,m,n	0.34 ± 0.34 (1.14)e,o,p	0.14 ± 0.21 (0.66)f,p,q,r	0.23 ± 0.57 (1.10)g,q,s	0.32 ± 0.46 (1.30)h,o,r,s

Data is expressed as mean ± SD (P95). Values in the same row showing different subscripts present a statistically significant difference ($p \leq 0.05$). Mann-Whitney U and Wilcoxon tests have been applied with Bonferroni correction. Comb., Combined nitroso compounds; *N*-NAs, *N*-nitrosamines; NDBA, *N*-Nitrosodibutylamine; NDEA, *N*-Nitrosodiethylamine; NDMA, *N*-nitrosodimethylamine; NDPA, *N*-Nitrosodi-n-propylamine; NMA, *N*-nitrosomethylaniline; NMEA, *N*-Nitrosomethylethylamine; NMOR, *N*-Nitrosomorpholine; NPIP, *N*-nitrosopiperidine; NPYR, *N*-nitrosopyrrolidine; NSAR, *N*-Nitrososarcosine; TCNA, sum of 10 carcinogenic *N*-NAs; TCNA with PF, sum of 10 carcinogenic *N*-NAs considering potency factor.

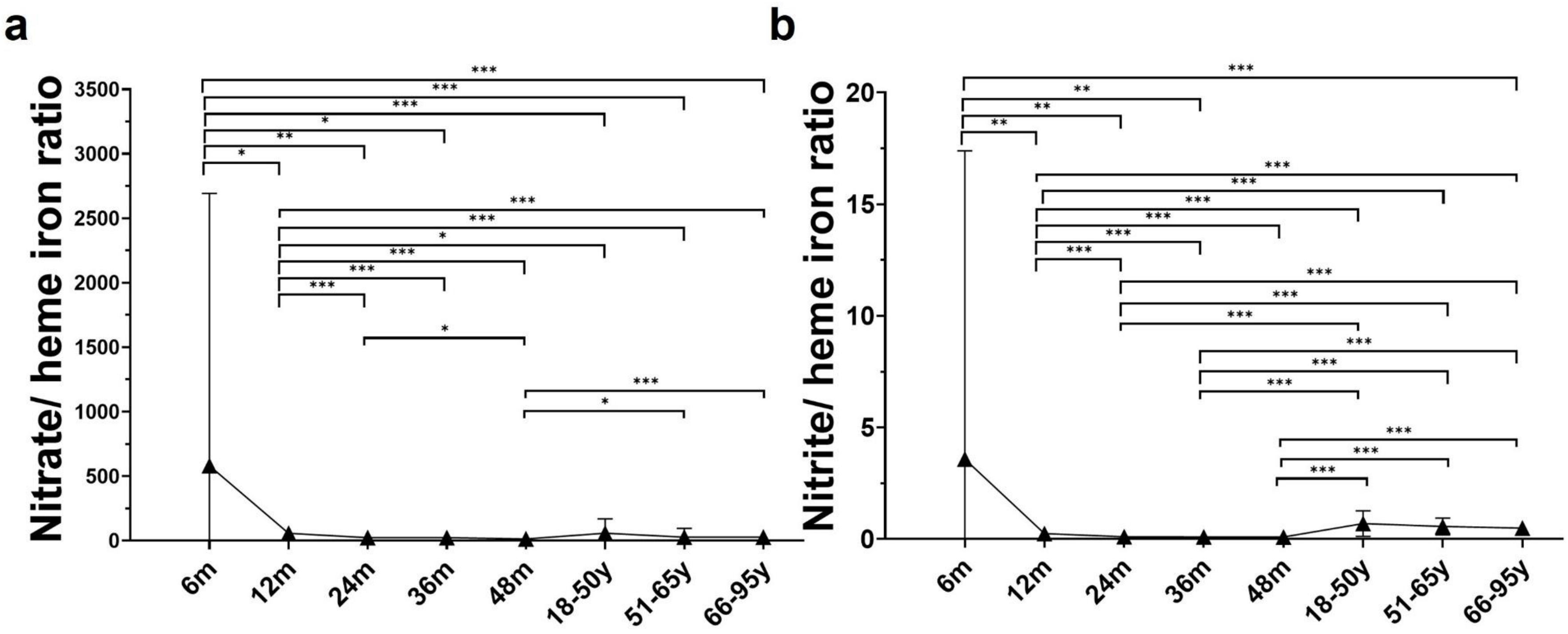

Supplementary Fig. S1. Dietary intake of precursors ratios of endogenous nitrosation across age groups. **a** Nitrate/ heme iron. **b** Nitrite/heme iron. Statistical significance was determined by Mann-Whitney U and Wilcoxon tests with Bonferroni correction (6m n= 112, 12m n= 109; 24m n= 93; 36m n=84; 48m n= 42; 18-50y n= 21; 51-65y n= 67; 66-95y n=94). * *p*-value < 0.05, ** *p*-value < 0.01, *** *p*-value < 0.001.

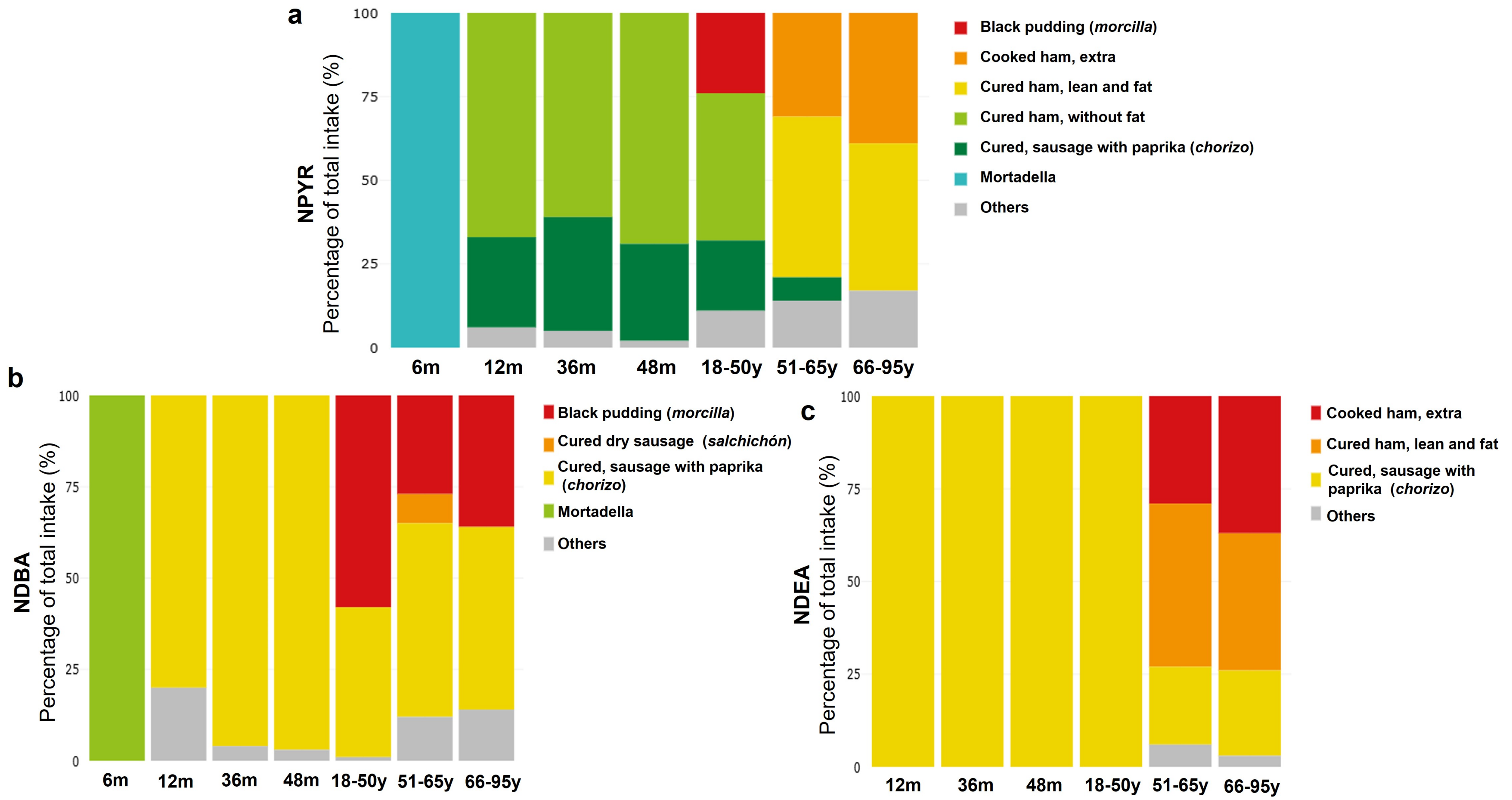

Supplementary Fig. S2. Dietary sources of minor *N*-NAs across age groups. **a** NPYR. **b** NDBA. **c** NDEA. *N*-NAs, *N*-nitrosamines; NDBA, *N*-Nitrosodibutylamine; NDEA, *N*-Nitrosodiethylamine; NPYR, *N*-nitrosopyrrolidine.

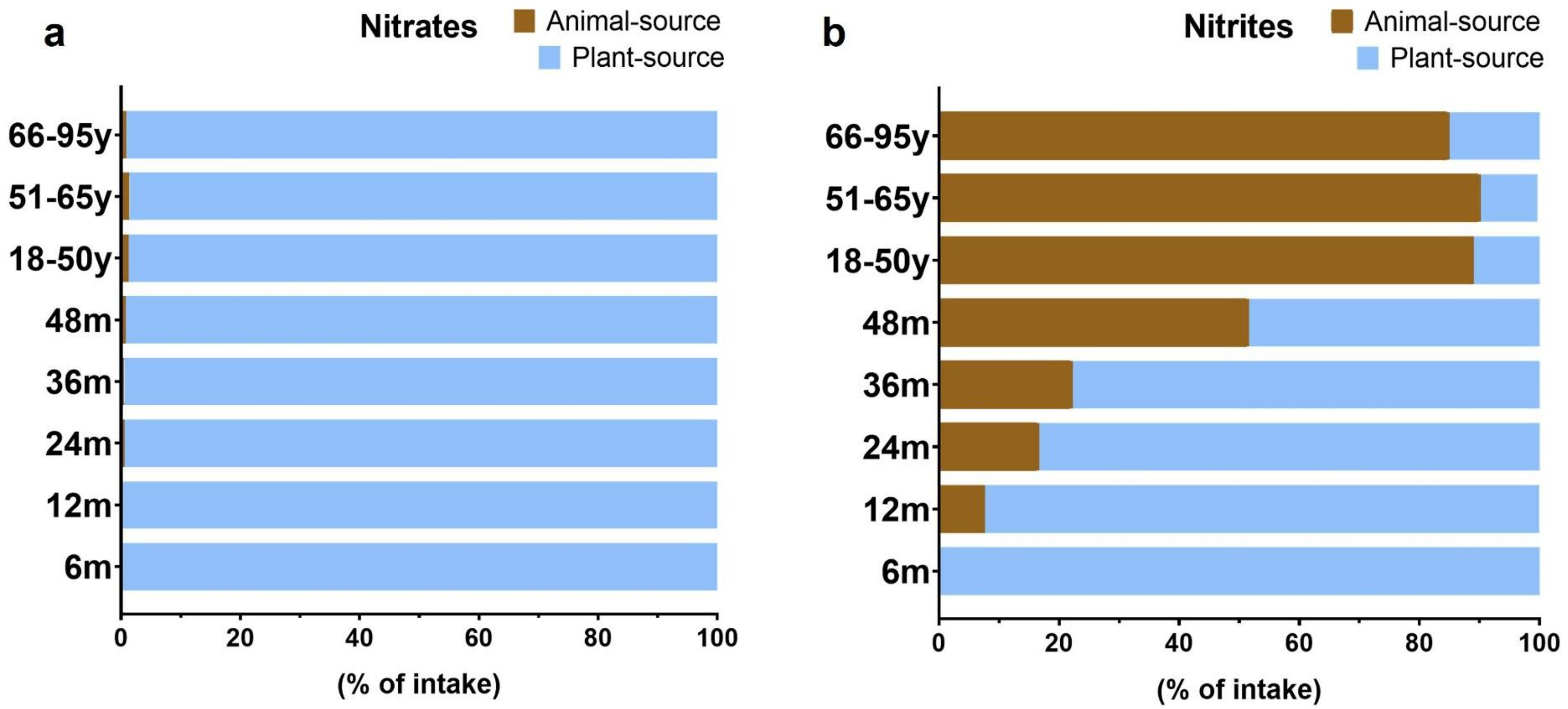

Supplementary Fig. S3. Contribution of animal and plant sources to the intake of precursors of *N*-NA formation across age groups. **a** Nitrates. **b** Nitrites.
